# Toxins, mutations and adaptations

**DOI:** 10.7554/eLife.66676

**Published:** 2021-02-23

**Authors:** Maarten De Jong, Neal M Alto

**Affiliations:** Department of Microbiology, University of Texas Southwestern Medical CenterDallasUnited States

**Keywords:** Burkholderia, type VI secretion system, evolution, bacterial warfare, *E. coli*, Other

## Abstract

The toxins that some bacteria secrete to kill off rival species can also generate mutations that help toxin-resistant populations adapt to new environments.

**Related research article** de Moraes MH, Hsu F, Huang D, Bosch DE, Zeng J, Radey MC, Simon N, Ledvina HE, Frick JP, Wiggins PA, Peterson SB, Mougous JD. 2021. An interbacterial DNA deaminase toxin directly mutagenizes surviving target populations. *eLife* **10**:e62967. doi: 10.7554/eLife.62967

Bacterial communities are often comprised of numerous different species which either co-exist in harmony or compete with each other for resources. To gain an upper hand on the competition, some bacteria have developed a form of needle-like machinery called a type VI secretion system (or T6SS for short) that injects toxic proteins directly into their rivals (Basler et al., 2012; Hood et al., 2010; Klein et al., 2020; Russell et al., 2011). It is generally thought that the toxins secreted by T6SS decapacitate the target organism by impairing important processes such as cell wall synthesis, ATP production and DNA replication (Ahmad et al., 2019; Jurėnas and Journet, 2020; Whitney et al., 2015). While these toxins are clearly involved in anti-bacterial warfare, it is unclear whether T6SS can also facilitate symbiotic relationships within bacterial communities.

Recently, a collaboration between groups led by Joseph Mougous (University of Washington) and David Liu (Harvard University and the Broad Institute) discovered that the T6SS of *Burkholderia cenocepacia* secretes a toxin called DddA that catalyzes the removal of an amino group from cytosine, converting it to uracil (Mok et al., 2020). In most cells, these enzymes – known as cytosine deaminases – are important for maintaining the levels of nucleotide precursors in cells (Neuhard, 1968). While most cytosine deaminases catalyze this reaction in single-stranded DNA, DddA is the first enzyme found to convert cytosine to uracil in double-stranded DNA. However, uracil is normally only found in RNA, where it pairs with another nucleotide base called adenosine: in DNA, adenosine pairs with thymine, whereas cytosine pairs with guanine in both DNA and RNA (Figure 1).

**Figure 1.**
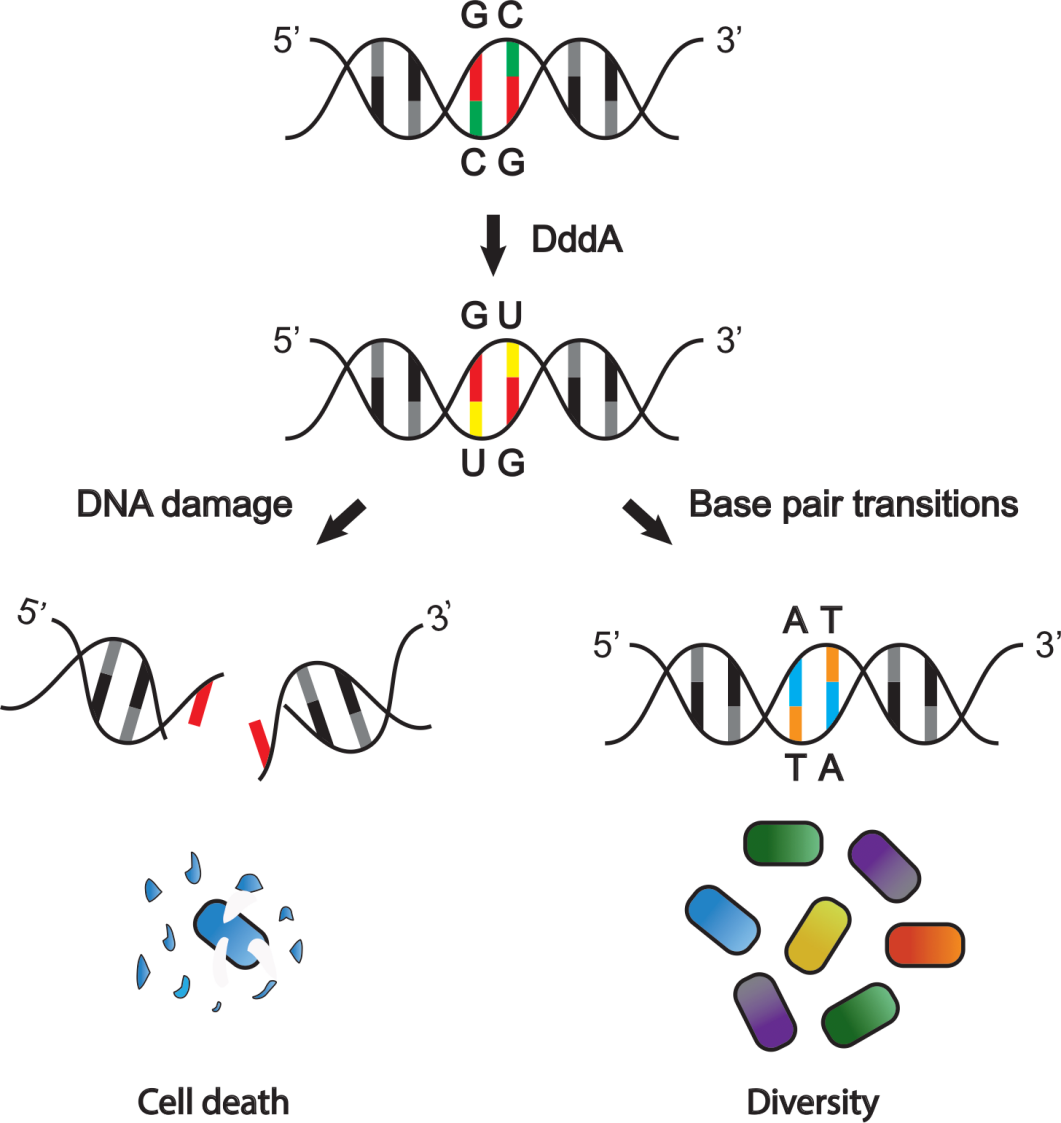
The DddA toxin from *B. cenocepacia* affects other bacterial species in different ways. DddA is a toxin that removes an amino group from cytosine (C; green), converting it into uracil (U; yellow) in chromosomal DNA (top). In some bacterial species uracil is then removed by the DNA repair machinery (left), which can lead to double-stranded DNA breaks and ultimately cell death. In bacteria resistant to the toxic effects of DddA, DNA breaks do not occur (right): instead, uracil is converted into thymine (T; orange), which causes guanine (G; red) to convert to adenosine (A; blue). This results in genetic variation within the targeted population, further diversifying the community of bacteria.

Now, in eLife, Mougous and colleagues – including Marcos de Moraes as first author – report that as well as destroying bacteria, DddA also provides a selective advantage for some bacteria in the community (de Moraes et al., 2021). The team (who are based at the University of Washington) found that when the bacterium *B. cenocepacia* injects DddA, uracil accumulates in the DNA of the targeted bacteria. Since uracil is normally only found in RNA, its presence triggers a repair mechanism that attempts to remove it from the DNA. However, this repair mechanism can lead to a break in one strand of the DNA, and if there are too many breaks in close proximity, they can lead to double-stranded breaks which stop DNA replication and result in bacterial cell death (Figure 1; D'souza and Harrison, 2003; Wallace, 2014). Indeed, de Moraes et al. found that the DddA delivered by *B. cenocepacia* suppresses the viability of several other bacterial species, including *Pseudomona aeruginosa* and *Burkholderia thailandensis*.

Although DddA causes double-stranded DNA breaks and cell death in most bacterial species, some disease-causing bacteria – including *Eschericia coli* and *Salmonella enterica –* are able to resist its detrimental effects. To better understand this observation, de Moraes et al. examined the long-term effects of DddA on these bacteria. They found that rather than excising the uracil that had replaced cytosine, the DNA replication machinery in the infected bacteria converts the uracil into a different nucleotide, thymine. As thymine pairs with adenosine in DNA, C-G pairs throughout the genome get replaced with T-A pairs (Figure 1).

An unanticipated consequence of this exchange was that the bacteria not killed by DddA acquired resistance to the antibiotic rifampicin. Further experiments revealed that other deaminase toxins similar to DddA were also able to introduce mutations in single-stranded DNA, suggesting this may be a widespread mechanism within bacterial populations (de Moraes et al., 2021).

It is generally thought that DNA mutations that arise during natural selection are caused by errors during chromosome replication or by exogenous factors such as chemicals and ionizing stress (Schroeder et al., 2018). Some bacteria rapidly adapt by importing fragments of foreign DNA from the environment and integrating them into their genome. This mechanism has been proposed to increase the genetic variation of populations, which provides a selective advantage to bacteria living in challenging environments or competing with other species (Dubnau and Blokesch, 2019; Mell and Redfield, 2014). The findings of de Moreas et al. suggest a similar mechanism in which the mutations produced by deaminase toxins help to diversify the population, creating new variants that can rapidly adapt to environmental changes.

There are several exciting avenues of research resulting from these findings. First, it is unclear why some bacteria are susceptible to DddA intoxication, whereas others are resistant. Defining these resistance mechanisms could help reveal the biological scenarios in which T6SS helps communities of bacteria adapt to their environment. Second, it will be interesting to determine how DddA toxins alter the composition of bacterial communities: DddA intoxication could eliminate competing species, while the genetic variations induced in resistant species may help enhance the long-term fitness of the community. Lastly, new insights into these topics may increase our understanding of human diseases where pathogenic bacteria come into contact with communities of microbes in the body, such as inflammatory bowel disease and cystic fibrosis, which could ultimately lead to better treatments (Buffie and Pamer, 2013; Spiewak et al., 2019).
